# Identification of pLG72-Induced Oxidative Stress Using Systemic Approaches

**DOI:** 10.1155/2015/429253

**Published:** 2015-10-11

**Authors:** Maofeng Wang, Hsi-Ju Chen, Jun Zhang, Weimin Li, Xinyou Xie, Hao-Teng Chang

**Affiliations:** ^1^Department of Clinical Laboratory, Sir Run Run Shaw Hospital, School of Medicine, Zhejiang University, Hangzhou, Zhejiang 310000, China; ^2^Department of Biomedical Sciences Laboratory, Affiliated Dongyang Hospital of Wenzhou Medical University, Dongyang, Zhejiang 322100, China; ^3^Graduate Institute of Basic Medical Science, College of Medicine, China Medical University, Taichung 40402, Taiwan; ^4^Department of Internal Medicine, Affiliated Dongyang Hospital of Wenzhou Medical University, Dongyang, Zhejiang 322100, China; ^5^Department of Computer Science and Information Engineering, Asia University, Taichung 41354, Taiwan

## Abstract

*G72* is a schizophrenia-susceptible gene encoding a polypeptide with 153 amino acids. In 2002, it was originally proposed as an activator of D-amino acid oxidase (DAOA) that could enhance the activity of DAAO and subsequently reduce the neurotransmission of *N*-methyl-D-aspartate receptors. However, several controversial findings have been reported recently. Due to a number of inconsistent descriptions of pLG72's biofunctions, this study aims to identify the cellular effects induced by pLG72 in U87 cells using systems biology approaches. The analyses of transcriptomics and biological networks showed that pLG72 might be involved in the induction of oxidative stress. To confirm the *in silico* prediction, we tested and discovered that overexpression of pLG72 effectively enhanced reactive oxygen species (ROS) in U87 cells and, furthermore, this induction can be quenched by Tempol, a general ROS scavenger. Therefore, *G72*-transgenic mice presenting some psychiatric symptoms, along with the pLG72 level being significantly increased in the serum of patients with schizophrenia, have led us to propose that the ROS enhancement in mental diseases may be from the overexpression of pLG72 in brain cells.

## 1. Introduction

*G72* is a gene encoding polypeptide with 153 amino acids in length, which was first identified as a schizophrenia-susceptible gene [1]. At the genetic level, people with the polymorphic variations of this gene have been determined to be susceptible to schizophrenia [2]. In addition to schizophrenia, the variations of* G72* gene have also been discovered to correlate with bipolar disorder [3, 4]. The most famous single nucleotide polymorphism (SNP), rs2391191 resulting in Arg30 to Lys, has been identified as having a correlation with the decreased thickness of the brain cortex in patients with schizophrenia [5]. This discovery also mentioned that the Arg30Lys could be a marker to determine the memory performance in patients with schizophrenia. Moreover, the Arg30 allele has previously been significantly associated with poorer episodic memory function [6]. This might indicate that pLG72 plays a role in the brain development or the fate of brain cells.

In 2002, Chumakov and colleagues reported that, in 213 patients with schizophrenia and 241 normal subjects in Canada, there was a highly susceptible locus in chromosome 13 which is correlated with schizophrenia. There was a gene named* G72* located in the susceptible region. Using yeast two hybrid experiments, the authors identified a pLG72-interacting partner D-amino acid oxidase (DAAO) physically. The DAAO activity was found to increase depending upon the increase of pLG72 concentration in a dose dependent manner. Therefore, pLG72 was firstly identified as a DAAO activator. They formulated the hypothesis that pLG72 is overexpressed in patients with schizophrenia so that DAAO would be overactivated, resulting in a decrease of D-serine and hypofunction of NMDA receptors in synapsis. Loss of brain and serum D-serine concentration has been recognized as a sign of schizophrenia [7]. However, in 2008, Kvajo and colleagues mentioned that in their experiments the interaction between pLG72 and DAAO could not be observed [8]. They firstly figured out that pLG72 located in mitochondria made mitochondrial dysfunction and fragmentation. This report raised another hypothesis that pLG72 can damage cells through damaging mitochondrial morphology. In addition to these two reports, a third group, Sacchi's group, has provided an opposite hypothesis, which is that pLG72 could inhibit DAAO activity in cells [9]. Based on their biochemical study, they assumed that the brain level of pLG72 is lower in patients with schizophrenia, resulting in the activation of DAAO and finally a decreased level of D-serine concentration in synapsis. Although our study demonstrated that the serum pLG72 level is higher in patients with schizophrenia [10] and supported the finding that pLG72 can activate DAAO [11], the function of pLG72 is still controversial. In this paper, we aim to predict the biological function of pLG72 by employing systems biology approaches and to demonstrate our hypothesis using cell biology methods.

## 2. Methods and Materials

### 2.1. Cell Culture

U87 cells were cultured in *α*-Minimum Essential Media (*α*-MEM) supplemented with 10% heat-inactivated fetal bovine serum at 37°C in an incubator comprising 5% CO_2_ and 95% air.

### 2.2. Transfection

The plasmids pcDNA4/TO/myc, His A (pcDNA4/TO) and pcDNA4/TO/myc, His A-*G72* (pcDNA4/TO/*G72*) were individually transfected into U87 cells using polyJet transfection reagent (polyJet, US). For the transfection, U87 cells were seeded onto a 100 mm culture plate. After 24 hr in culture (~80–90% confluency), the 5 *μ*g of plasmid DNA and 15 *μ*L of polyJet reagent were mixed in serum-free *α*-MEM. The transfection mixture was gently vortexed and incubated for 15 min at room temperature to allow the formation of transfection complexes. The transfection mixture was then added dropwise to the cells and incubated at 37°C for 16 hr. The medium-containing transfection mixture was replaced using fresh complete medium, and after another 48 hr the cells were harvested for RNA isolation, Western blotting, or flow cytometry analyses. The transfection efficiency was tested greater than 40% using pEGFP-C1 plasmids as the reporter plasmid.

### 2.3. Total RNA Isolation

The U87 cells were seeded in a 100 mm plate and transfected with pcDNA4/TO and pcDNA4/TO/*G72* separately. At 48 hr after transfection, total RNA was extracted using an RNA Isolation Kit (GeneMark, Taiwan) according to the manufacturer's instructions. The concentration and purity of the RNA were measured by a NanoDrop 1000 spectrophotometer (Thermo Fisher Scientific, USA). Purity was checked by the ratio of the OD260/OD280 and OD260/OD230. The quality of total RNA was accessed using an Agilent 2100 Bioanalyzer (Agilent Technologies, USA) [12].

### 2.4. Human Oligonucleotide DNA Microarray (HOA)

The Human Whole Genome One Array version 6 (Phalanx BiotechGroup, Taiwan) contains 32,679 DNA oligonucleotide probes, and each probe is a 60-mer designed in the sense direction. Among the total number of probes, 31,741 correspond to the annotated genes in the RefSeq v51 and Ensembl version 65 database. In addition, 938 control probes are also included. The detailed descriptions of the gene array list are available from http://www.phalanx.com.tw/attachment/download/HOA/HOA6_probe_sequence.zip [12].

### 2.5. Microarray Analysis

Fluorescent aRNA targets were prepared from 1 *μ*g total RNA samples using a One Array Amino Allyl aRNA Amplification Kit (Phalanx Biotech Group, Taiwan) and Cy5 dye (GE Healthcare, US). Fluorescent targets were hybridized to the Human Whole Genome One Array with Phalanx hybridization buffer using the Phalanx Hybridization System. After 16 hr hybridization, nonspecific binding targets were washed away. The slides were then scanned using a DNA Microarray Scanner (Model G2505C, Agilent Technologies, USA). The Cy5 fluorescent intensities of each spot were analyzed by GenePix 4.1 software (Molecular Devices, USA).

Each single sample was at least performed twice in terms of technical or biological replicates under a reproducibility of more than 0.975. The signal intensity was loaded into Rosetta Resolver System (Rosetta Biosoftware, USA) to do data preprocessing and applied to 75 percentile centering normalization. The errors of the sample were estimated by using an error-weighted approach at the same time. Both fold change and *p* value for pairwise sample comparisons were calculated for evaluating differentially expressed genes. In our implementation, the spots with *p* < 0.05 and *R* ≥ 1 were identified as differentially expressed genes for further pathway analysis. The DNA microarray data has been submitted to and approved by the Gene Expression Omnibus Database (GEO) with an accession number of GSE67704.

### 2.6. GeneMANIA and NOA Analysis

The genes upregulated by more than 2-fold and with the statistic *p* of less than 0.05 were selected and assembled for a gene expression profile (Table 1). The differentially expressed genes were separated into three subdatasets: upregulated, downregulated, and total gene. These three sets were input into GeneMANIA (http://www.genemania.org) [13, 14] individually using the default setting. Because no significant prediction was presented, the parameters regarding GO functions were selected manually one by one.

The NOA (http://app.aporc.org/NOA) database was designed for identifying the enrichment of gene ontology based on biological networks as classified by systems biology [15]. The three subdatasets, upregulated, downregulated, and total differentially expressed genes, were input into and analyzed by the NOA server to determine the relationship of network ontology.

### 2.7. Measurement of ROS Level

U87 cells were transfected with pLG72 overexpression plasmids as described previously. After 48 hr, the medium was removed and the cells were treated with or without 10 mM of Tempol, a ROS scavenger, at 37°C for 1 hr. After removal of the medium, cells were incubated with fresh serum-free medium containing 20 *μ*M 2′,7′-Dichlorofluorescein Diacetate (DCFH-DA, Sigma, US). The cells were incubated at 37°C for 30 min and then the cells were washed, trypsinized, and finally analyzed using flow cytometry, BD FACSCanto (BD Biosciences, USA) with excitation at 488 nm and emission at 526 nm.

U87 cells treated with or without 100 *μ*M of H_2_O_2_ were set as positive and negative controls, respectively, to ensure ROS generation.

## 3. Results

### 3.1. Differential Gene Expression of Microarray Analysis

*G72* is a human specific gene susceptible to psychiatric disorders. This gene is specifically expressed in glial cells in the brain; however, there has been no evidence of probed pLG72* in situ*. Thus, a glial cell line U87 was employed in this study. The cells were transfected with pLG72 overexpression plasmids, and the total RNA was isolated at 48 hr after transfection. After DNA microarray analysis, transcriptomics of pLG72 overexpression identified 65 genes with more than a 2-fold differential change as compared with the control, in which 27 genes were upregulated and 38 were downregulated as shown in the gene expression profile (Table 1). Looking at the functions of these genes, there are 15 located in plasma or the mitochondrial membrane, including 3 ion channels (PANX2, CLCN2, and NPHS2), 7 receptors (RTN1, ACVR2B, CHRNA6, EFNB1, NTRK2, EDNRA, and NR2C1), and 5 membrane-integrated proteins (TM4SF18, SCUBE2, TOMM20L, OTOG, and TMEM216). Usually, membrane proteins, especially receptors, can receive extracellular stimulation and transact the signals to intracellular molecules. In the pLG72 overexpression profile, there are 6 genes involved in signaling transduction (RTN1, CNTD2, MAP4K4, HIPK3, TRIM40, and SAG) and 6 genes participating in calcium binding and secondary messenger transduction (GALNT3, TBC1D8B, SCUBE2, SMOC2, EDNRA, and PLA2G5). According to the analyses above, pLG72 may regulate intracellular response through transducing several phosphorylation and secondary messenger signaling. Because of this, another high throughput technique, micro-Western blotting, must be employed to screen the signaling regulatory pathway transduced by pLG72 overexpression. In addition, in the transcriptomic profile of pLG72 overexpression, there are 8 genes classified as nucleotide binding proteins (HNRNPCL1, FOXR2, ADAL, MAF, UPP1, ARL8A, NR2C1, and PSMA1). Nucleotide binding proteins are involved in many biological processes such as nucleotide synthesis and metabolism, energy exchange, and DNA/RNA synthesis. This interesting finding can be further investigated in the future.

### 3.2. Network Construction with the Differentially Expressed Genes

**Figure 1:**
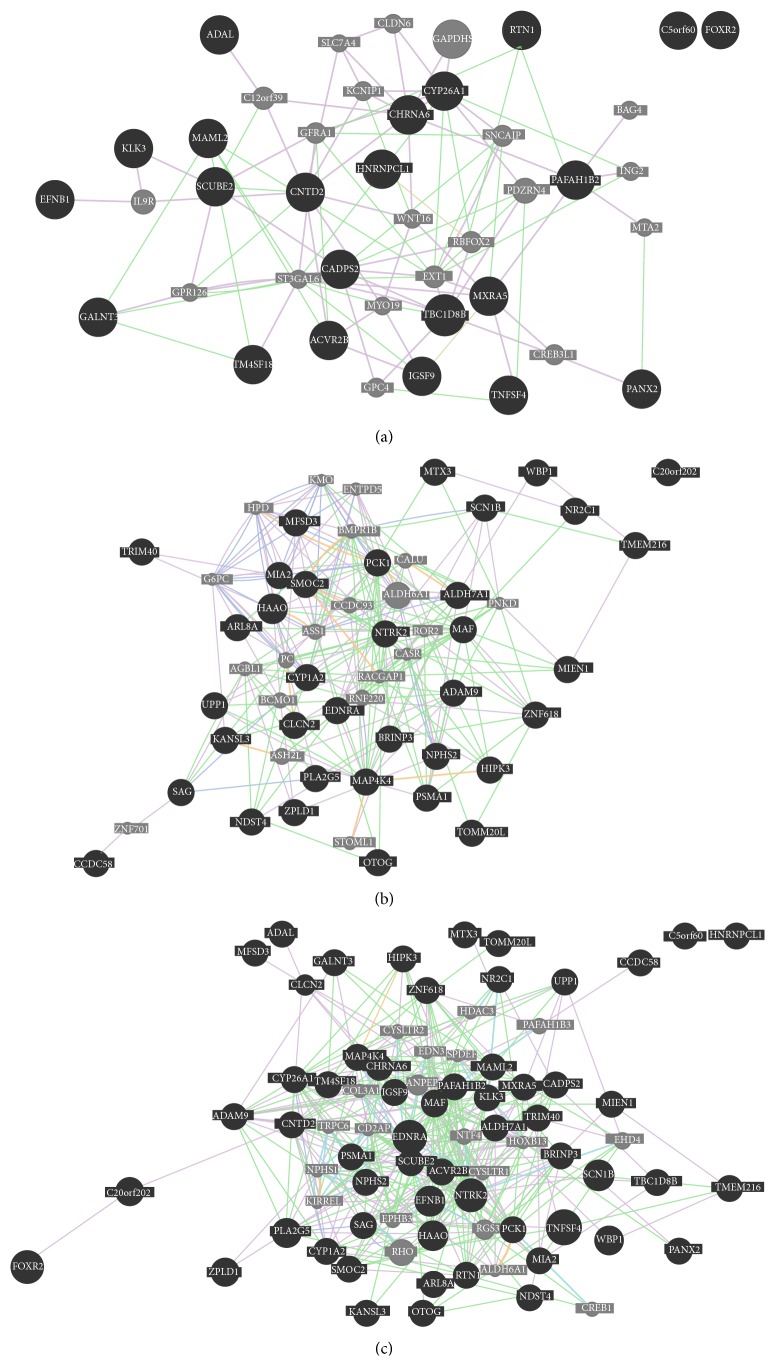
GeneMANIA analysis. The upregulated genes (a), downregulated genes (b), and total differentially expressed genes (c) were input into GeneMANIA server using the default setting.

In order to investigate the biological functions/processes induced by pLG72 overexpression, the network generation tool, GeneMANIA, was employed. GeneMANIA was developed for biofunction predictions of favorite genes or gene sets based on Gene Ontology annotation patterns. GeneMANIA finds the genes related with a given input gene from public datasets including protein-protein interaction, genetic regulation, pathways, reactions, gene coexpression, protein colocalization, protein domain similarity, and phenotypic screening profiles. Additional genes to the input gene will be presented to make a complete and comprehensive network. The 27 genes upregulated and 38 genes downregulated by pLG72 overexpression were separately input into the GeneMANIA system (Figures 1(a) and 1(b)). To generate a more complete network, a total of 65 differentially expressing genes were also input into the GeneMANIA system (Figure 1(c)). The system parameters of GeneMANIA were set to their default. Although GeneMANIA presented a comprehensive network, no predictive biological functions can be given automatically. However, when the parameter of biological processes is selected manually, an oxidation-related genetic pathway can be proposed.

### 3.3. Network Ontology Analyses (NOA)

The biological networks are usually used for prediction of biological pathways, annotation of biological functions, and/or identification of targets of diseases. Since GeneMANIA only gave a low-confidence clue of oxidative reaction, NOA, which applied the categories of Gene Ontology (GO) to network analysis [16], was employed to emphasize the effects induced by pLG72 overexpression. Three GO categories separately listed GO terms according to their *p* value significance. The top GO term in biological process revealed that pLG72 might increase the production of oxidative stress via hydrogen peroxide synthesis (Table 2, bold). In addition, nitric oxide biosynthesis and metabolism and nitric oxide synthase activity also were predicted in the biological process and molecular function categories, respectively (Table 2, italic). Moreover, arginine metabolic process in the biological process and arginine binding in molecular function were predicted and might function to produce nitrogen oxide and enhance ROS [17] (Table 2, underlined). In the molecular function category, “tetrahydrobiopterin binding” is the first ranking of the prediction (Table 2). According to Gene Ontology definition, “tetrahydrobiopterin binding” can inference the activity of tetrahydrobiopterins which are enzyme cofactors that carry electrons in redox reactions. Therefore, NOA provides a highly confident prediction to posit that pLG72 might induce oxidative stress in U87 cells.

### 3.4. Demonstration of Reactive Oxygen Species (ROS) Induction

**Figure 2:**
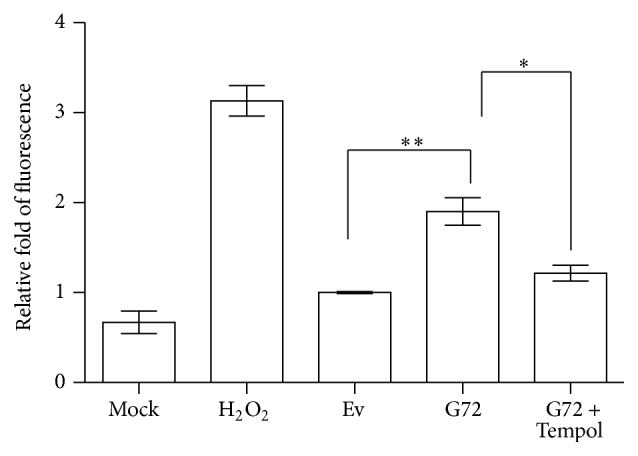
The measurement of ROS production. The ROS production was detected using a fluorescent indicator, DCFH-DA, and monitored by flow cytometry. The ROS level was significantly induced by pLG72 and scavenged by Tempol. Ev, empty vector;  ^*∗*^ *p* < 0.05;  ^*∗∗*^ *p* < 0.01.

To demonstrate the induction of oxidative stress proposed above, U87 cells were transfected with pLG72 overexpression plasmids and the intracellular ROS was stained using DCFH-DA, the general ROS indicator. The fluorescent intensity was measured using flow cytometry. As compared with the control group of empty vector (EV), ROS level significantly increased 69.2% in U87 cells transfected with* G72* plasmids (Figure 2). This indicates that pLG72 overexpression could raise oxidative stress via increasing the level of ROS production. To further confirm ROS production, Tempol, a general ROS scavenger with a superoxide dismutase mimetic, was employed. Figure 2 shows that when Tempol was added, the level of ROS raised by pLG72 overexpression was significantly decreased to a level similar to that of U87 cells transfected with empty vector. That means the oxidative stress induced by pLG72 overexpression might be derived from superoxide overproduction.

## 4. Discussion

In 2013, Cheng and colleagues reported that 361 genes were differentially expressed in the brains of* G72* transgenic mice, some of which were implicated in neurological or psychological disorders [18]. However, they did not mention the possible mechanisms for the enhanced psychiatric behaviors of* G72* transgenic mice, including fewer stereotypic movements in the open field test, higher baseline startle responses in the course of the PPI test, and lower hedonic responses in the sucrose preference test. After the comparison between our gene expression profile and Cheng's, genes in the two individual datasets implied that the oxidative stress might be induced by pLG72 overexpression and we subsequently have proposed that although the cell viability showed no significant difference, the increase of oxidative stress might be involved in long-term damage of brain cells causing dysregulation of neurotransmission and/or neuron death. Of course, since the gene expression profiles in this study were provided by transient transfection using pLG72 expression plasmids, it would be very different from the transcriptomics data from Cheng's* G72*-transgenic mice* in vivo* owing to the 40% transient transfection efficiency. Our data was limited to provide the functional information of pLG72 protein in U87 cells.

In 2011, Otte and colleagues discovered that in* G72* transgenic mice the activity of complex I in the respiratory chain was attenuated, presumably through the binding between the flavin-mononucleotide (FMN) group and pLG72 [19]. The impaired complex I increased superoxide (O_2_^−∙^) production, which in turn led to reduced aconitase activity and increased production of lipid/protein peroxidation. Furthermore, treatment with* N*-acetyl cysteine, a precursor of glutathione (GSH), increased the antioxidant capacity and rescued the spatial learning deficit [19]. On the other hand, pLG72 interacts with cytosolic DAAO and then blocks the translocation of DAAO into peroxisomes which metabolize H_2_O_2_ to H_2_O [9]. The enzymatic reaction of DAAO will produce H_2_O_2_ as a side product. Therefore in U87 cells pLG72 might enhance oxidative stress* via* the interaction of DAAO and blockage of the DAAO peroxisome targeting, resulting in producing H_2_O_2_ in cytosol and/or mitochondria.

Cappelletti's and Sacchi's groups also transfected pLG72 into U87 cells [20, 21]. Sacchi and colleagues found that pLG72 interacted with DAAO in mitochondria, and Cappelletti and colleagues reported overexpression pLG72 could increase the turnover of DAAO to avoid excessive D-serine depletion in brain. In their paper, they also mentioned that the half-life of pLG72 is short only 25 min–40 min. Therefore, how this short turnover protein can significantly increase oxidative stress is valuable to be investigated. With what is told in this study, pLG72 might play a role in neurological disorders, especially psychiatric diseases, in the way it damages neuronal cells slowly through the enhancement of mitochondrial ROS levels.

## Figures and Tables

**Table 1:** The differential expression affected by pLG72 overexpression in U87 cells.

Gene symbol	Fold change	*p* value	Gene symbol	Fold change	*p* value	Gene symbol	Fold change	*p* value
DAOA^a^	99.9	<2.80*E* − 45^b^	TBC1D8B	6.4	3.250*E* − 09	C5orf60	2.8	1.508*E* − 42
HNRNPCL1	22.2	3.287*E* − 31	CADPS2	5.7	5.697*E* − 40	ADAL	2.8	3.527*E* − 26
FOXR2	14.4	<2.80*E* − 45	LINC00028	4.7	5.597*E* − 05	SCUBE2	2.8	4.477*E* − 32
PANX2	11.7	7.113*E* − 39	IGSF9	3.8	0.000550514	CYP26A1	2.6	5.026*E* − 18
GALNT3	10.9	<2.80*E* − 45	TNFSF4	3.6	<2.80*E* − 45	PAFAH1B2	2.5	5.294*E* − 21
RTN1	10.1	<2.80*E* − 45	MXRA5	3.6	1.051*E* − 10	CHRNA6	2.5	4.314*E* − 09
LOC100287063	10.0	6.157*E* − 32	HSPA1B	3.4	6.485*E* − 16	KLK3	2.4	2.067*E* − 26
TM4SF18	7.3	0.001431557	LOC100130705	3.1	<2.80*E* − 45	EFNB1	2.1	1.061*E* − 06
CNTD2	5.6	6.912*E* − 08	ACVR2B	3.0	<2.80*E* − 45			
RN7SK	6.1	8.507*E* − 34	MAML2	3.0	1.129*E* − 16			

NCF1B	−24.3	<2.80*E* − 45	UPP1	−2.8	1.229*E* − 14	HIPK3	−2.6	6.306*E* − 44
WBP1	−16.4	8.353*E* − 32	ALDH7A1	−2.7	5.883*E* − 19	NR2C1	−2.6	0.000500818
TOMM20L	−5.7	<2.80*E* − 45	CLCN2	−2.7	4.905*E* − 33	SCN1B	−2.6	0.00099985
ZNF618	−5.7	5.221*E* − 20	ADAM9	−2.7	1.640*E* − 07	PSMA1	−2.6	2.490*E* − 18
LOC100133251	−3.8	1.984*E* − 23	CYP1A2	−2.7	2.620*E* − 35	TMEM216	−2.6	1.675*E* − 31
MAF	−3.5	3.682*E* − 05	MFSD3	−2.7	1.343*E* − 16	PLA2G5	−2.6	8.703*E* − 24
ZPLD1	−3.2	1.677*E* − 18	NDST4	−2.7	9.920*E* − 17	KANSL3	−2.5	1.840*E* − 13
OTOG	−3.0	5.315*E* − 16	HAAO	−2.7	2.481*E* − 13	TRIM40	−2.5	1.702*E* − 06
SMOC2	−3.0	1.154*E* − 08	ARL8A	−2.6	3.177*E* − 27	SAG	−2.5	5.333*E* − 11
CCDC58	−3.0	5.483*E* − 05	C20orf202	−2.6	6.332*E* − 06	FAM5C	−2.5	3.283*E* − 09
NTRK2	−3.0	1.381*E* − 25	EDNRA	−2.6	2.281*E* − 15	PCK1	−2.4	7.419*E* − 11
MIEN1	−2.9	5.327*E* − 31	MTX3	−2.6	9.082*E* − 06	NPHS2	−2.3	4.093*E* − 10
MAP4K4	−2.8	1.432*E* − 23	MIA2	−2.6	4.305*E* − 20			

^a^ *DAOA* is *G72*. It will not be included in the following analysis.

^b^The minimal *p* value calculated using Rosetta Resolver System is 2.80*E* − 45.

**Table 2:** The upregulated genes analyzed with Network Ontology Analysis.

Gene ontology	*p* value	Term name
Biological process	4.3*E* − 4	**Hydrogen peroxide biosynthetic process**
	4.3*E* − 4	Arginine catabolic process
	8.4*E* − 4	*Nitric oxide biosynthetic process*
	0.0010	*Nitric oxide metabolic process*

Cellular component	0.0080	Cytoplasmic cyclin-dependent protein kinase holoenzyme complex
	0.0080	Cell outer membrane
	0.0162	Subsynaptic reticulum
	0.0201	Spindle midzone

Molecular function	4.5*E* − 5	*Tetrahydrobiopterin binding*
	4.5*E* − 5	*Nitric-oxide synthase activity*
	9.0*E* − 5	Arginine binding
	9.3*E* − 4	Steroid hormone receptor activity
